# Prediction of circRNAs Based on the DNA Methylation-Mediated Feature Sponge Function in Breast Cancer

**DOI:** 10.3389/fbioe.2019.00365

**Published:** 2019-11-26

**Authors:** Yue Gu, Ce Ci, Xingda Zhang, Mu Su, Wenhua Lv, Chuangeng Chen, Hui Liu, Dongwei Zhang, Shumei Zhang, Yan Zhang

**Affiliations:** ^1^College of Bioinformatics Science and Technology, Harbin Medical University, Harbin, China; ^2^Department of Breast Surgery, Harbin Medical University Cancer Hospital, Harbin, China; ^3^School of Life Science and Technology, Harbin Institute of Technology, Harbin, China; ^4^Department of General Surgery, The Second Affiliated Hospital of Harbin Medical University, Harbin, China; ^5^College of Information and Computer Engineering, Northeast Forestry University, Harbin, China; ^6^State Key Laboratory of Respiratory Disease, Guangzhou Medical University, Guangzhou, China

**Keywords:** circular RNA, DNA methylation, miRNA sponge, breast cancer, network

## Abstract

Several studies have found that DNA methylation is associated with transcriptional regulation and affect sponge regulation of non-coding RNAs in cancer. The integration of circRNA, miRNA, DNA methylation and gene expression data to identify sponge circRNAs is important for revealing the role of DNA methylation-mediated regulation of sponge circRNAs in cancer progression. We established a DNA methylation-mediated circRNA crosstalk network by integrating gene expression, DNA methylation and non-coding RNA data of breast cancer in TCGA. Four modules (26 candidate circRNAs) were mined. Next, 10 DNA methylation-mediated sponge circRNAs (sp_circRNAs) and five sponge driver genes (sp_driver genes) in breast cancer were identified in the CMD network using a computational process. Among the identified genes, *ERBB2* was associated with six sponge circRNAs, which illustrates its better sponge regulatory function. Survival analysis showed that DNA methylations of 10 sponge circRNA host genes are potential prognostic biomarkers in the TCGA dataset (*p* = 0.0239) and GSE78754 dataset (*p* = 0.0377). In addition, the DNA methylation of two sponge circRNA host genes showed a significant negative correlation with their driver gene expressions. We developed a strategy to predict sponge circRNAs by DNA methylation mediated with playing the role of regulating breast cancer sponge driver genes.

## Introduction

Breast cancer is one of the most common malignant tumors in women (Koboldt et al., 2012). The HER2 (encoded by *ERBB2*) amplification group has achieved great clinical success because HER2 is an effective therapeutic target for breast cancer (Slamon et al., 1987). However, the current targeted therapy for breast cancer has not been fully and effectively implemented (Ayca and Traina, 2011). Studies have shown that dysregulated gene expression in breast cancer is affected by various genetic and epigenetic factors, including frequent somatic mutations (Nik-Zainal et al., 2016), copy number variations (Nik-Zainal et al., 2016), single nucleotide polymorphisms (SNPs) (Michailidou et al., 2017), non-coding RNA (Iorio et al., 2015), and DNA methylation (Yang et al., 2001). Abnormal regulation of epigenetic modifications leads to aberrant gene expression and function (Berger et al., 2009; Pan et al., 2017). Epigenetic modifications thus play an important role in the development and progression of cancer (Esteller, 2008).

DNA methylation is a common epigenetic modification that regulates gene expression at the transcriptional level (Lindahl, 1981; Bird, 1984; Baylin and Jones, 2011; Wise and Charchar, 2016). Abnormal DNA methylation, including global hypomethylation and aberrant methylation status on key regulatory elements, has been observed in multiple human cancers (Trimarchi et al., 2011; Aran et al., 2013). Many studies have found significant differences in DNA methylation between breast cancer samples and normal samples. In general, cancer cells show hypomethylation of the entire genome, while promoter sequences of tumor suppressor genes show hypermethylation. Hypomethylation of the whole genome is caused by genomic instability and loss of gene imprinting, which causes overexpression of oncogenes (Trimarchi et al., 2011). In contrast, hypermethylation of tumor suppressor gene promoters causes genetic silencing of tumor suppressor genes (Bird, 1996; Aran et al., 2013). The hypomethylation of the gene promoter CpG island promotes its downstream gene transcription, whereas its hypermethylation inhibits the transcription of the downstream genes (Deaton and Bird, 2011).

Circular RNAs (circRNAs) form a covalently linked continuous loop and lack 5′ and 3′ ends and polyadenylation tail structures (Diener, 1989; Chen and Yang, 2015; Song et al., 2016). CircRNAs exhibit important effects on RNA-related protein binding, gene splicing regulation and transcription, as well as the modification of parental gene expression (Li et al., 2015; Peng et al., 2017). Recent studies have shown that circRNAs are more stable than their linear counterparts and some function as biological markers for disease treatment (Weil et al., 2015; Zhang et al., 2015; Abu and Jamal, 2016; Kulcheski et al., 2016). Many studies have shown that circRNAs can also function as miRNA sponges in the regulation of gene expression (Hansen et al., 2013; Jens, 2014; Qiang et al., 2016). It can be involved in the development of a variety of diseases, such as atherosclerotic disease, prion disease, and myotonic dystrophy (Qu et al., 2015). For example, the circular transcript CDR1as (or ciRS-7), which is encoded by the reverse genome of the human CDR1 gene in human and mouse brain tissues, functions as an miRNA sponge to miR-7(Hansen et al., 2011). ciRS-7 inhibits miR-7 activity and competes with miR-7 in binding to other RNAs to modulate target gene expression (Hansen et al., 2013; Peng et al., 2015). Several studies have demonstrated that circRNAs also exhibit functions in cancer. For instance, circ-Amotl1 is highly expressed in breast cancer samples and many cancer cell lines (Yang et al., 2017). Interactions between circ-Amotl1 and c-myc lead to increased tumorigenicity (Yang et al., 2017). The potential clinical value of circRNAs in cancer diagnosis, therapy and prognosis has been the subject of recent investigation (Abu and Jamal, 2016; Kulcheski et al., 2016; Yang et al., 2017).

circRNAs are weakly expressed and have a long half-life (Enuka et al., 2016). The detection process of circRNA expression is technically challenging (Enuka et al., 2016). Here we integrated genome, epigenome and non-coding RNA data of breast cancer samples and used a bioinformatics approach to identify potential important sponge circRNAs in breast cancer. This study demonstrates the important role of circRNA sponge regulation associated with DNA methylation in breast cancer, which suggests a therapeutic strategy for manipulating the driver gene function in breast cancer through circRNA sponge regulation.

## Materials and Methods

### Data Sources

miRNASeq data (1,102 breast cancer samples, 104 normal samples), Illumina Infinium Human Methylation 450 BeadChip level 3 data (684 cancer samples, 96 normal samples), and RNASeqV2 data (1,102 breast cancer samples, 113 normal samples) were downloaded from The Cancer Genome Atlas (TCGA) (https://gdc.cancer.gov/). Clinical data were downloaded from TCGA (https://gdc.cancer.gov/) and GSE78754. Among them, DNA methylation and gene expression matching data were found in 781 cancer samples and 84 normal samples. CircRNA-miRNA interaction data were downloaded from the Starbase v2.0 (http://starbase.sysu.edu.cn/). MiRNA-target gene interaction data were downloaded from miRTarBase (http://mirtarbase.mbc.nctu.edu.tw/), which stores experimentally verified miRNA-target gene interaction information. CircRNA location information was obtained from Circbase (http://www.circbase.org/). The protein coding driver gene list was derived from Vogelstein et al. (Vogelstein et al., 2013). The reference genome (hg19) was obtained from UCSC (http://genome.ucsc.edu/).

### Data Preprocessing

We integrated miRNASeq data from 1,102 breast cancer samples and 104 normal samples to construct miRNA expression profiles between breast cancer samples and normal samples. If there was a missing value for miRNA expression, the miRNA was removed.

We used Illumina Infinium Human Methylation 450 BeadChip data from 684 breast cancer samples and 96 normal samples and circRNA position information to construct the DNA methylation profile of circRNA host genes. We performed data preprocessing to remove probes containing missing values in the sample and probes for multiple genes, CpG sites on the sex chromosomes and SNPs. Previous studies showed that multiple adjacent CpG sites have the same DNA methylation pattern (Lehmann-Werman et al., 2016); if a circRNA host gene contains multiple CG loci, the DNA methylation level of the circRNA host gene is quantified as the average methylation level of these CG loci.

We used the same samples to construct the DNA methylation profile of target genes interacting with differentially expressed miRNAs. We performed data preprocessing to remove probes containing missing values in the sample and probes for multiple genes, CpG sites on the sex chromosomes and SNPs. The DNA methylation of target gene was quantified as the average methylation level of CG loci located in the target gene.

We use RNASeqV2 data of breast cancer samples and normal samples to construct target gene expression profile interacting with differentially expressed miRNAs. We removed target genes with missing values.

### Establishment of the DNA Methylation-Mediated circRNA Crosstalk (DMCC) Network and Identification of Candidate circRNAs

First, differentially expressed miRNAs (fold change > 2 or fold change < 0.5, *q* value < 1) were screened between breast cancer samples and normal samples using SAMR package. CircRNAs that interacted with differentially expressed miRNAs were matched. If two circRNAs shared the same miRNA (two circRNAs have sequence and function similarity), then both circRNAs form a connection. A circRNA crosstalk (CC) network was constructed.

In addition, the DNA methylation profile of circRNA host genes in the CC network was constructed according to the data preprocessing description. Differential methylation circRNA host genes between breast cancer samples and normal samples were screened by SAMR package (fold change > 1.5 or fold change < 2/3, *q* value < 5), and Pearson correlation of DNA methylation of two circRNA host genes was calculated. If the Pearson correlation of two circRNA host genes was greater than random (permutation > 1,000) even >0.6, these two circRNA host genes will be two nodes of one edge in the CC network. The DMCC network was constructed with Pearson correlation as the weight. The circRNA host genes are co-methylated in the DMCC network.

Module-mining was performed in the DMCC network by using MCODE plugin in the Cytoscape, and circRNAs in the modules were selected as the candidate circRNAs.

### miRNA Target Prediction and circRNA-miRNA-Driver (CMD) Gene Network Establishment

All human miRNAs and target information were derived from the miRTarBase database, which stores the experimental validated miRNA targets and is prevalent in the target prediction. We used SAMR package to screen differentially expressed genes between breast cancer samples and normal samples (fold change > 2 or fold change < 0.5, *q* value < 1). We obtained breast cancer driver genes by taking the intersection of breast cancer differentially expressed genes and cancer driver genes. We used candidate circRNAs, breast cancer driver genes and all shared miRNAs to construct the CMD network. Each pair of circRNA-driver gene in this network is a candidate circRNA-driver gene pair. All implementations of network diagrams and module mining are realized by Cytoscape.

### Predicting the Sponge circRNAs of Protein-Coding Driver Genes

Studies have shown that circRNAs are almost co-expressed with their linear transcripts (Enuka et al., 2016). Therefore, DNA methylation of the circRNA host genes may regulate the expression of circRNAs, which influences the competitive regulation of circRNAs as miRNA sponge. Similarly, DNA methylation of breast cancer driver genes can also regulate the expression of driver genes. Sponge circRNAs positively regulate the expression of their targets, and their regulation depends on the miRNA stoichiometry (Du et al., 2016). Therefore, we used the following hypothesis to predict sponge circRNA-driver gene pairs: sponge circRNA host genes and target driver genes are co-methylated and they interact with the same miRNAs.

A complete computational analysis process was designed to predict sponge circRNA-driver gene pairs. First, for each candidate circRNA-driver gene pair, the significance (*P*_1_: *P*-value of Fisher's exact test) of shared miRNA with same seeds and the significance (*P*_2_: *P*-value of Pearson's correlation coefficient test) of the DNA methylation correlation between a circRNA host gene and driver gene promoter region (−1.5 to +0.5 kb relative to transcription start site) in breast cancer samples were calculated. We computed *P*_1_ using formula (1):

(1) $$\begin{array}{l} P_{1}=\frac{C_{a+b}^{a}C_{c+d}^{c}}{C_{n}^{a+c}}=\frac{\left(a+b\right)!\left(c+d\right)!\left(a+c\right)!\left(b+d\right)!}{a!b!c!d!n!} \end{array}$$

where *a* is the number of shared miRNAs in a candidate circRNA-driver gene pair; *b* is the number of shared miRNAs in a circRNA-other driver genes; *c* is the number of shared miRNAs in a driver gene-other circRNAs; *d* is the number of remaining miRNAs; and *n* represents the number of total miRNAs.

We computed *P*_2_ using formula (2):

(2) $$\begin{array}{l} P_{2}=P\left(r\right) \end{array}$$

with

(3) $$\begin{array}{l} r=\frac{1}{n-1}\overset{n}{\underset{i=1}{\sum }}\left(\frac{X_{i}-\bar{X}}{s_{X}}\right)\left(\frac{Y_{i}-\bar{Y}}{s_{Y}}\right) \end{array}$$

where X is the DNA methylation profile of circRNA host genes in breast samples, and Y is the DNA methylation profile of driver genes in breast cancer samples.

The following conditions were fulfilled for the candidate circRNA-driver gene pairs as predicted sponge circRNA-driver gene pairs: (1) *P*_1_ and *P*_2_ are no larger than a threshold of 0.05 (*P*_1_ ≤ 0.05 and *P*_2_ ≤ 0.05); (2) the Pearson correlation of DNA methylation between circRNA host genes and driver gene promoter region shows a positive correlation and greater than random (permutation 1000 times, *p* < 0.05); (3) circRNA-driver gene pairs that shared at least eight unique targeting sites for these shared miRNAs (different miRNAs sharing the same seed sequence could target the same site) are selected (Du et al., 2016); and (4) there was a significant negative correlation between DNA methylation of driver gene promoter region and the expression of driver genes (*p* < 0.05).

### Functional Enrichment Analysis of circRNA Host Genes Associated With DNA Methylation

The circRNA host genes in the DMCC network are associated with DNA methylation and are co-methylated. We next analyzed the function of circRNA host genes in the DMCC network. Functional and pathway enrichment of the DNA methylation-mediated circRNA host genes was achieved using DAVID (https://david.ncifcrf.gov/). Rich factor is the ratio of the number of circRNA host genes mapped to this GO term and the number of annotated genes in this term; the higher rich factor means the more significant enrichment. *P*-value was corrected by Benjamini. Enrichment analysis. The graph was performed using the ggplot2 package in R 3.2.1.

### Survival Analysis

The clinical survival data for breast cancer were downloaded from TCGA and GEO (GSE78754). Survival data of breast cancer patients were integrated (missing data were removed). Finally, we obtained 656 breast cancer samples in TCGA and 70 breast cancer samples in GSE78754. We assessed the predictive effect of sponge circRNAs on overall survival in breast cancer by survival analysis. Survival analysis was performed using Cox proportional hazards model. DNA methylation of the sponge circRNA host gene was used as a covariate to assess the independent contribution of each circRNA host gene to prognosis. The DNA methylation value of the circRNA host gene was multiplied by the linear sum of the regression coefficients in the multivariate Cox regression to assign a prognostic index (PI). PI for each patient was calculated as shown in formula 4.

(4) $$\begin{array}{l} \text{PI}=\beta_{1}x_{1}+\beta_{1}x_{1}+\ldots +\beta_{n}x_{n} \end{array}$$

where *x*_*i*_ represents DNA methylation of circRNA host gene i; β_*i*_ represents regression coefficient of gene i in multivariate Cox regression; and n represents the number of genes. The median of PI was used as a threshold to classify breast cancer patients into the high risk group and low risk group. We used R V3.2.1 for the survival analysis and generating survival plot.

## Results

### Differential Target Gene Expression and DNA Methylation of circRNA Host Genes and Target Genes in Breast Cancer

**Figure 1:**
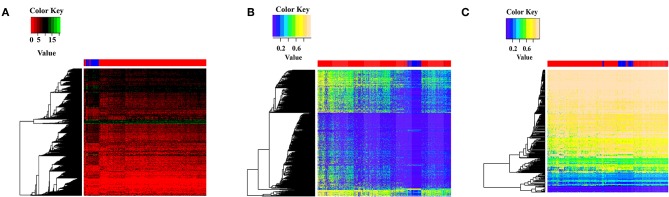
Feature display of target genes and circRNAs that interact with differentially expressed miRNAs. **(A)** The target genes expression of differential expression miRNAs between breast cancer samples and normal samples. Gene expression values were log2 transformed after adding a pseudo-value of 1 to avoid infinite values. Red indicates breast cancer samples, blue indicates normal samples. **(B)** DNA methylation cluster analysis of target genes bound by differentially expressed miRNAs. Red represents breast cancer samples, blue represents normal samples. **(C)** DNA methylation cluster analysis of circRNA host genes that interact with differentially expressed miRNAs. Red indicates breast cancer samples, and blue indicates normal samples.

miRNAs are abundantly present in many organisms and can selectively interact with complementary mRNAs to reduce protein production. We identified 192 differentially expressed miRNAs between breast cancer samples and normal samples in TCGA datasets. To determine potential targets of these miRNAs, we next used miRTarBase, which revealed 4,546 potential target genes of the differentially expressed miRNAs. Notably, the target genes also showed differential expression between breast cancer and normal samples and target gene expression can separate breast cancer samples from normal samples (Figure 1A). This suggests that these differentially expressed miRNAs in breast cancer may be targeting specific mRNAs to regulate target gene expression, leading to the differential gene expression in breast cancer samples.

We next constructed a DNA methylation profile of the 4,546 target genes between 684 breast cancer samples and 96 normal samples. The DNA methylation heat map showed that the target genes showed differential methylation between breast cancer and normal samples, with higher DNA methylation in breast cancer compared with normal samples. Furthermore, DNA methylation of the target genes could separate breast cancer samples and normal samples (Figure 1B). These findings suggest that the differential levels of DNA methylation may impact target gene expression in breast cancer.

circRNA can bind to miRNAs through miRNA response elements (MREs) and regulate miRNA functions. We found that 747 circRNAs showed potential interaction with the 192 differentially expressed miRNAs. DNA methylation of circRNA host genes may affect their miRNA sponge function and impact miRNA target gene expression. To explore the potential role of DNA methylation of circRNA host genes, we constructed a DNA methylation profile of the 747 circRNA host genes and generated a DNA methylation heat map. The heat map showed that circRNA host genes showed differential methylation between breast cancer samples and normal samples, and the DNA methylation of circRNA host genes could separate breast cancer samples from normal samples (Figure 1C). These results confirm differential DNA methylation of circRNA host genes in breast cancer and suggests that methylation of circRNA host genes may indirectly regulate target gene expression.

### Construction of the DMCC Network and Identification of Candidate circRNAs

**Figure 2:**
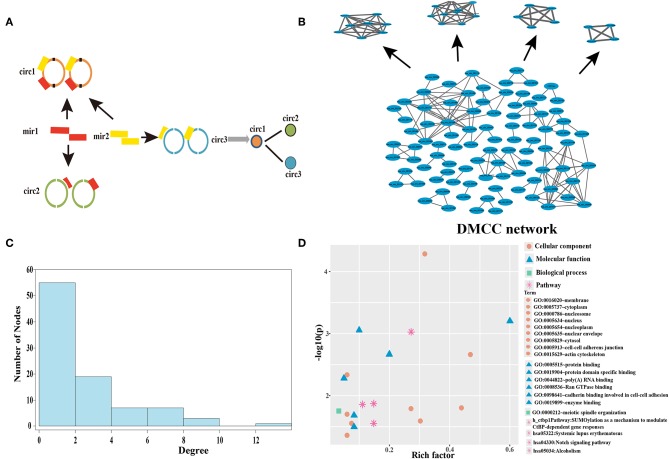
Establishment and analysis of DMCC network in the breast cancer. **(A)** The construction of the circRNA crosstalk (CC) network. The orange, green, and blue represent three different circRNAs, and the red and yellow rectangles are two miRNAs. **(B)** The DNA methylation mediated circRNA crosstalk (DMCC) network and candidate circRNAs. The blue nodes represent circRNAs. **(C)** Degree distribution of DMCC network. **(D)** The function and pathway enrichment analysis of circRNA host genes in the DMCC network. The abscissa represents the rich factor, and the ordinate represents the inverse of the logarithm of the *P*-value corrected by Benjamini. The larger value represents the significant of GO terms and pathway.

We next aimed to predict sponge circRNAs from the perspective of epigenetics regulation. To first identify circRNAs that highly correlated with DNA methylation, we established a DMCC network. We screened the 192 miRNAs differentially expressed in breast cancer and found that they formed 2,419 circRNA-miRNA interactions. A circRNA crosstalk (CC) network (Supplementary Figure 1) was established by sharing same miRNAs (Figure 2A), thus two circRNAs have sequence similarity. The CC network contained 747 circRNAs and 48,492 circRNA-circRNA connections. The circRNA-circRNA network represents the interactions between circRNAs, in which two circRNAs may have sequence functional similarities. The maximum degree of the CC network is 938. The degree distribution of the CC network is subject to the power-law distribution (Supplementary Figure 2), and therefore the CC network is a biologically significant network.

We next constructed a DNA methylation profile of the 747 circRNA host genes according to the circRNA location information and identified 201 differentially methylated circRNA host genes (*P* < 0.05). DNA methylation of these circRNA host genes could separate cancer samples from normal samples (Supplementary Figure 3). We calculated the Pearson correlation coefficient of DNA methylation between every two circRNA host genes. By connecting the two circRNAs with a Pearson correlation coefficient greater than random even >0.6 (*P* < 0.05), a DMCC network was constructed (Figure 2B). The circRNA host genes in the DMCC network are characterized by co-methylation. The DMCC network contains 92 circRNAs and 138 circRNA-circRNA interactions. The degree distribution of the DMCC network is also subject to the power-law distribution (Figure 2C), and therefore the DMCC network is also biologically significant.

Our results above show that the 92 circRNAs in the DMCC network are differentially methylated in breast cancer samples, with sequence similarity and co-methylation characteristics. We next evaluated the biological function of these circRNA host genes by DAVID and found that the circRNA host genes are mainly enriched in the cell membrane, cytoplasm, protein binding, and protein domain specific binding (Figure 2D). These genes are mainly enriched in SUMOylation as a mechanism to modulate CtBP-dependent gene responses, systemic lupus erythematosus, and the Notch signaling pathway (Figure 2D).

To identify novel circRNAs associated with DNA methylation, module mining in the DMCC network was performed. Four network modules (Figure 2B) were obtained in the DMCC network according to the module score. Four circRNAs (hsa_circ_000582, hsa_circ_002025, hsa_circ_002024, hsa_circ_001851) are seed nodes of four modules. A total of 26 circRNAs in the four modules were used as candidate circRNAs for subsequent studies.

### Establishment and Global Properties of the CMD Network

miRNAs interact with circRNAs as well as cancer driver genes through MREs (Bartel, 2009; Lü et al., 2017). Thus, some circRNAs may act as sponges by binding to miRNAs and preventing their interactions with cancer driver genes. These circRNAs therefore regulate the expression of driver genes and play a very important role in disease regulation.

**Figure 3:**
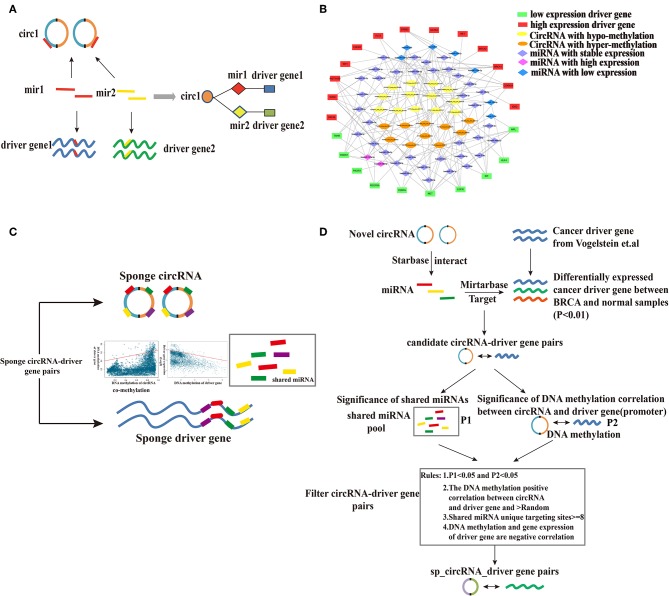
Establishment of the CMD gene network and prediction process of sponge circRNA-driver gene pairs. **(A)** The construction of the CMD gene network. **(B)** The CMD network. The ellipse represents circRNAs, the diamond represents miRNAs, and the rectangle represents driver genes. **(C)** Construction of sponge regulatory mechanism for circRNA and driver gene by targeting the same miRNA and prediction of sponge circRNAs. **(D)** Computational strategy of predicting sponge circRNA-protein coding driver genes in breast cancer.

To examine the regulatory interactions among potential circRNAs, their miRNA targets and putative cancer driver genes, we established a CMD gene network. We first screened 4,541 differentially expressed genes between breast cancer and normal samples (fold change > 2 or fold change < 0.5, *p* < 0.01). Differentially expressed genes and 125 cancer driver genes were selected and 25 breast cancer driver genes were obtained. According to rules (Figure 3A), we constructed a CMD network (Figure 3B), containing 23 circRNAs, 23 cancer driver genes and 45 miRNAs. The CMD network is divided into three layers: driver genes act as functional layer in the outermost layer, and the internal circRNAs indirectly regulate driver genes through miRNAs in the middle layer.

The miRNA-mediated complex CC network was established by the circRNA-miRNA-driver gene interaction. The crosstalk type between the circRNA-miRNA-driver genes represents a complex transcriptional regulatory network. This provides a perspective on how to indicate the intermolecular relationship of cell behavior. Each circRNA-driver gene pair in the circRNA-miRNA-driver gene network served as candidate circRNA-driver gene pair for subsequent study.

### Prediction of Sponge circRNAs Regulating Cancer Driver Genes Based on the CMD Network

We next used a bioinformatics approach to predict sponge circRNA-driver gene pairs in breast cancer affected by DNA methylation.

Sponge circRNAs have a positive regulatory effect on their target gene expression (Hansen et al., 2011; Peng et al., 2015; Zheng et al., 2016), and the strength of their regulation depends on the stoichiometry of the involved miRNAs (Du et al., 2016). If the host gene of the sponge circRNA is hypermethylated, the circRNA host gene will be silenced, eliminating its ability to bind miRNAs, leading to downregulation of the target gene. This suggests that high methylation of host genes may correlate with downregulated target gene expression and vice versa. Therefore, the sponge circRNA may have the same methylation pattern as the sponge driver gene (Figure 3C).

**Figure 4:**
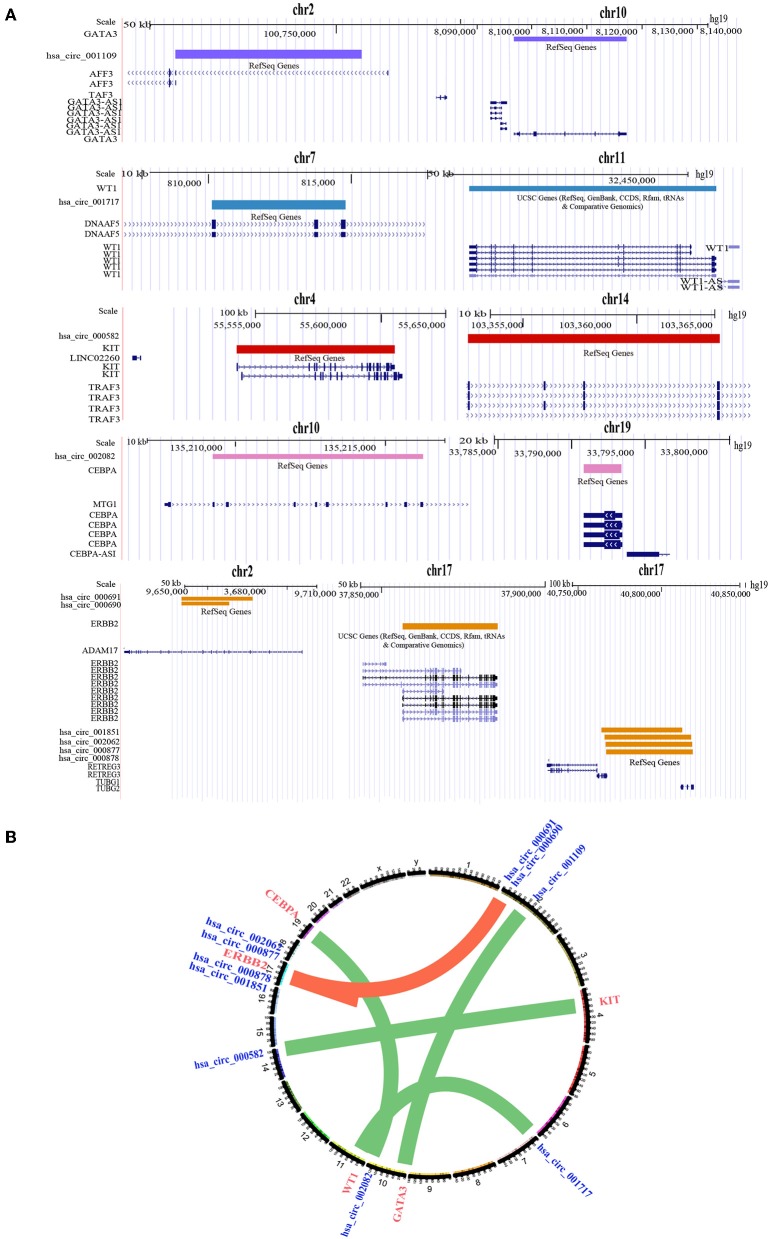
Location information of sponge circRNA-driver gene pairs. **(A)** Genome position information of 10 sponge circRNA-driver gene pairs. Purple bar represents the genome location of *GATA3* and its sponge circRNA. Blue bar represents the genome location of *WT1* and its sponge circRNA. Red bar represents the genome location of *KIT* and its sponge circRNA. Pink bar represents the genome location of *CEBPA* and its sponge circRNA. Yellow bar represents the genome location of *ERBB2* and its sponge circRNAs. **(B)** Sponge circRNA-driver gene pairs in breast cancer. The nodes represent circRNAs and driver genes (blue represents circRNAs, red represents driver genes) and the edges represent the predicted regulation between sponge circRNA and the corresponding protein-coding driver gene.

Based on this assumption, we developed a computational process to predict the sponge circRNA and gene pairs in breast cancer. CircRNA-breast cancer driver gene pairs that share at least one miRNA were selected as candidate circRNA-driver gene pairs. Using the prediction algorithm in the flow (Figure 3D), we identified 10 sponge circRNA-driver gene pairs comprising 10 sponge circRNAs and five protein-coding driver genes (Figures 4A,B).

We found that most of the sponge circRNAs and driver genes are located on the different chromosomes (even when located on the same chromosome, the distance was >2 Mb). This illustrates that the regulation between sponge circRNAs and sponge driver genes is a trans-acting relationship, supporting the mechanism by which sponge circRNAs indirectly regulate sponge driver gene expression by interacting with miRNAs.

### Characteristics of Sponge circRNAs Based on DNA Methylation

**Figure 5:**
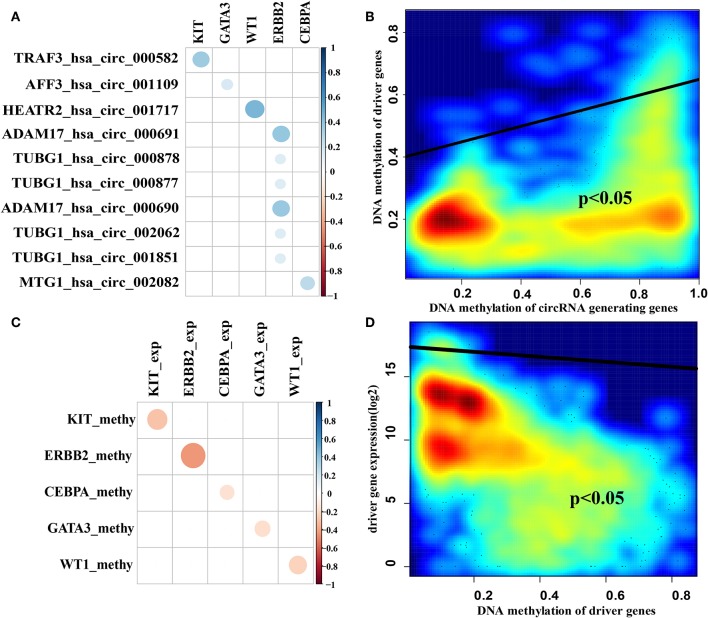
Correlation of sponge circRNAs and target driver genes. **(A)** The correlation coefficient diagram of DNA methylation of sponge circRNA host genes and target driver genes in breast cancer samples. **(B)** The smoothscatter plot of DNA methylation of sponge circRNA host genes and DNA methylation of target driver genes in breast cancer samples. **(C)** The correlation coefficient diagram of DNA methylation of target driver genes and sponge driver genes expression in breast cancer samples. **(D)** The smoothscatter plot of DNA methylation of sponge driver genes and sponge driver genes expression in breast cancer samples. Gene expression values were log2 transformed after adding a pseudo-value of 1 to avoid infinite values. The scalable size of circles in correlation coefficient diagram represent the absolute value of the correlation coefficient.

Many studies have shown that circRNAs can act as sponge circRNAs to competitively bind to miRNAs and regulate gene expression (Leonardo Salmena et al., 2011; Hansen et al., 2013; Zheng et al., 2016; Peng et al., 2017). We next analyzed the predicted sponge circRNA-driver gene pairs that showed the following characteristics: (1) DNA methylation of the sponge circRNA host gene was positively correlated with DNA methylation of the sponge driver gene promoter region in breast cancer samples (Figures 5A,B) and (2) DNA methylation of the driver gene promoter region was negatively correlated with driver gene expression in breast cancer samples (Figures 5C,D). We found that the *ERBB2* gene was associated with a large number of predicted sponge circRNAs compared with other examined genes (Supplementary Table 1), which suggests that it may have the better sponge regulation. We also examined DNA methylation of sponge circRNA host genes, DNA methylation of sponge driver genes and sponge driver gene expression patterns in breast cancer samples and normal samples (Supplementary Figure 5). The box plot showed that the DNA methylation of the sponge circRNA host genes and the DNA methylation of the sponge driver genes have the same tendency, while the DNA methylation of the sponge driver gene and sponge driver gene expression showed the opposite trend.

We further analyzed pathways of the predicted five cancer driver genes and found that *KIT, ERBB2* and *CEBPA* are related to PI3K and RAS pathways (Vogelstein et al., 2013). *GATA3* is associated with transcriptional regulatory core pathways (Vogelstein et al., 2013). *WT1* is associated with the chromosome modification core pathway (Vogelstein et al., 2013). These genes are enriched in the process of cell survival and cell fate (Supplementary Table 2) (Vogelstein et al., 2013). Together these results suggest that the driver genes regulated by sponge circRNAs may be associated with cancer. These results are consistent with previous studies that showed that sponge RNAs may have the same carcinogenic or tumor suppressor function as their regulatory genes (Du et al., 2016).

### Sponge circRNAs Are Potential Prognostic Biomarkers for Breast Cancer

To determine whether the sponge circRNAs are associated with the prognosis of breast cancer, we performed survival analysis using Infinium Human Methylation 450 BeadChip data and clinical survival data of breast cancer in TCGA and GSE78754.

**Figure 6:**
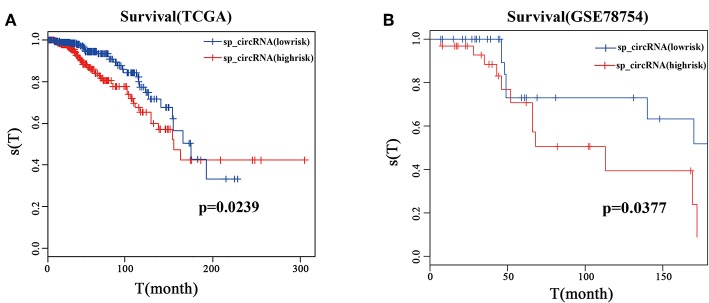
Survival analysis of 10 sponge circRNAs. **(A)** Survival analysis curve of 10 sponge circRNAs using TCGA clinical data (breast cancer). Red curve represents high risk patients, and blue curve represents low risk patients. **(B)** Survival analysis curve of 10 sponge circRNAs using GSE78754. Red curve represents high risk patients, and blue curve represents low risk patients.

Survival analysis shows that patients in the high risk group have worse overall survival than those in the low risk group in the TCGA dataset (Figure 6A, *p* = 0.0239). DNA methylation of the circRNA host genes clearly separated the high and low risk groups in breast cancer patients in the first 10 years. Similarly, patients in the high risk group showed worse survival compared with those in the low risk group in the GSE78764 dataset (Figure 6B, *p* = 0.0377), and DNA methylation of circRNA host genes separated the high and low risk groups of breast cancer patients. These results indicate that the 10 sponge circRNAs can not only be used as potential diagnostic markers for breast cancer, but also may reflect the prognosis of patients.

### DNA Methylation of circRNA Host Gene and Driver Gene Synergistically Affect Driver Gene Expression

**Figure 7:**
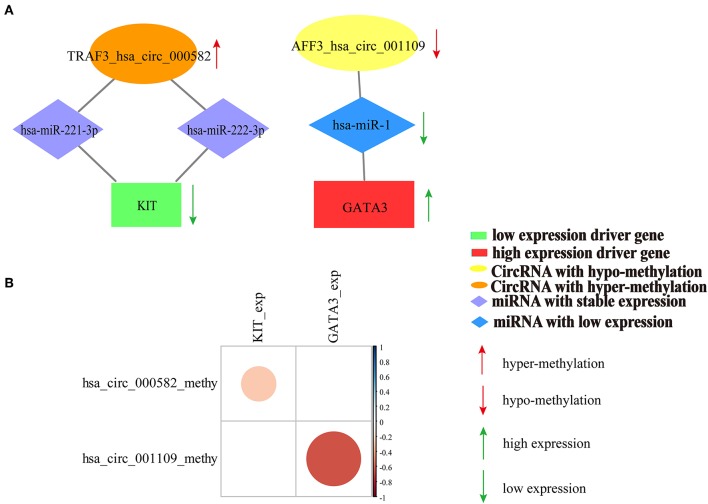
Relationship between the DNA methylation of the predicted two sponge circRNAs (hsa_circ_000582, hsa_circ_001109) and expression of their sponge driver genes (*KIT, GATA3*). **(A)** Competition interaction diagram of the predicted two sponge circRNAs (hsa_circ_000582, hsa_circ_001109) and their sponge driver genes (*KIT, GATA3*). **(B)** The correlation coefficient diagram of DNA methylation of the predicted two sponge circRNAs (hsa_circ_000582, hsa_circ_001109) and expression of their sponge driver genes (*KIT, GATA3*). The scalable size of circles in correlation coefficient diagram represent the absolute value of the correlation coefficient.

We also calculated the correlation between DNA methylation of circRNA host genes and driver genes with driver gene expression. The results indicated that DNA methylation of sponge circRNA host gene and DNA methylation of driver gene synergistically affect driver gene expression in breast cancer (Supplementary Figure 4). Moreover, DNA methylation of the predicted two sponge circRNAs (hsa_circ_000582, hsa_circ_001109) showed a significant negative correlation with the expression of their sponge driver genes (*KIT, GATA3*) (Figures 7A,B). The relationship between the expression of *ERBB2, CEBPA*, and *WT1* and DNA methylation of their sponge circRNAs is shown in Supplementary Figure 6.

## Discussion

CircRNAs are a class of non-coding RNAs that regulate gene expression at both transcriptional and post-transcriptional levels. Recent studies have shown that circRNAs can also function as miRNA sponges (Ebert and Sharp, 2010; Leonardo Salmena et al., 2011). CircRNAs play an important role in cancer and can be used as biological markers for prognosis.

In our study, we consider the effect of transcriptional and post-transcriptional levels on breast cancer. First, genomic, epigenetic genomics, and non-coding RNA data were integrated. Next, we constructed a DMCC network and a CMD gene network. We designed a computational process to predict sponge circRNA-driver gene pairs in breast cancer to evaluate the effect of DNA methylation of sponge circRNA host genes on circRNA sponge function. The regulation of sponge circRNA on the protein coding driver genes is not one to one, but there exists a mixed regulation. *ERBB2* showed a large number of predicted sponge circRNAs compared with other examined genes, indicating that *ERBB2* may have better circRNA sponge regulation function. ERBB2 is a 185 kDa cell membrane receptor encoded by the oncogene erbB-2, a member of the epidermal growth factor receptor family. Higher Ras-MAPK and PI3K-Akt signaling activity is detected in ERBB2 overexpressing tumor cells, which show stronger cell proliferation ability. Survival analysis showed that DNA methylation of sponge circRNA host genes could significantly differentiate breast cancer samples in two data sets. These results suggest that the predicted sponge circRNAs can be used as a prognostic biomarker for breast cancer.

The experimental methods that are currently used to verify sponge circRNAs require extensive time and are costly. Therefore, using bioinformatics calculation methods to identify sponge circRNAs may be more advantageous. While our study focused on predicting sponge circRNAs in breast cancer, these methods can be extended to other cancer types. We provide a new approach to study the regulatory effects of sponge circRNAs associated with DNA methylation in cancer, which may be helpful for understanding of the mechanism of competitive endogenous RNAs (ceRNA).

Our research reveals a complex DNA methylation-mediated circRNA sponge regulatory mechanism (Supplementary Figure 7). Interestingly, four of the 10 Sponge circRNAs (hsa_circ_000582, hsa_circ_000691, hsa_circ_000877, hsa_circ_000690) were identical to the breast cancer-specific circRNAs from previous studies (Coscujuela Tarrero et al., 2018). Sponge circRNAs may lead to abnormal expression of important protein coding driver genes in breast cancer. Furthermore, the DNA methylation-mediated disruption of circRNA sponge regulation may be a useful target for cancer treatment.

The quantification of the DNA methylation of circRNA host genes in this study is based on the Illumina Infinium HumanMethylation 450 BeadChip of breast cancer in the TCGA database, which covered only 482,421 CpG sites, whereas WGBS covers more CpG sites. Therefore, using high throughput sequencing data such as WGBS to quantify the DNA methylation of circRNA host genes may be more accurate, but still shows the problem of insufficient sample size.

Our research reveals a complex DNA methylation-mediated regulation of circRNA sponges. Sponge circRNAs bind to miRNAs to prevent their interactions with target genes, and sponge circRNAs showed the same DNA methylation pattern and expression pattern as the sponge-driven genes. Sponge circRNAs may significantly contribute to the abnormal expression of important protein-coding genes in breast cancer. The DNA methylation-mediated circRNA sponge regulation may be a potential target for cancer therapy. Our findings also show a new perspective of circRNA as a miRNA sponge in the pathogenesis.

## Data Availability Statement

Publicly available datasets were analyzed in this study. This data can be found here: https://gdc.cancer.gov/GSE78754.

## Ethics Statement

The studies involving human participants were reviewed and approved by the Ethics Committee of the working institution and in accordance with the Helsinki Declaration (revised in Fortaleza, Brazil, October 2013). The patients/participants provided their written informed consent to participate in this study. Written informed consent was obtained from the individual(s) for the publication of any potentially identifiable images or data included in this article.

## Author Contributions

YG, CCi, and SZ contributed to the study design, identification of the level of DNA methylation of circular RNA, analysis of data and manuscript draft. YZ contributed to the study design and supervision. XZ provided help about the mechanism of circRNA during the manuscript revision. MS, WL, CCh, and HL were responsible for data search and figure beautification. DZ contributed to the study supervision.

### Conflict of Interest

The authors declare that the research was conducted in the absence of any commercial or financial relationships that could be construed as a potential conflict of interest.
