# Supplementary material for: Prediction of circRNAs Based on the DNA Methylation-Mediated Feature Sponge Function in Breast Cancer
Source: Front Bioeng Biotechnol. 2019 Nov 26;7:365. doi: 10.3389/fbioe.2019.00365 (PMC6988805; doi:10.3389/fbioe.2019.00365)
Supplement: Supplementary file 1 [file Data_Sheet_1.PDF]

Supplementary material

Supplementary Table 1. The number of predicted sponge circRNAs for cancer driver gene

Driver gene	Number of sponge circRNAs
KIT	1
ERBB2	6
CEBPA	1
GATA3	1
WT1	1

Supplementary Table 2. The core pathway and process information of sponge driver gene

Gene Symbol	Gene Name	Core pathway	Process
KIT	v-kit Hardy-Zuckerman 4 feline sarcoma viral oncogene homolog	PI3K;RAS;STAT	Cell Survival
GATA3	GATA binding protein 3	Transcriptional Regulation	Cell Fate
WT1	Wilms tumor 1	Chromatin Modification	Cell Fate
ERBB2	v-erb-b2 erythroblastic leukemia viral oncogene homolog 2	PI3K;RAS	Cell Survival
CEBPA	CCAAT/enhancer binding protein (CEBP), alpha	PI3K;RAS; MAPK	Cell Survival

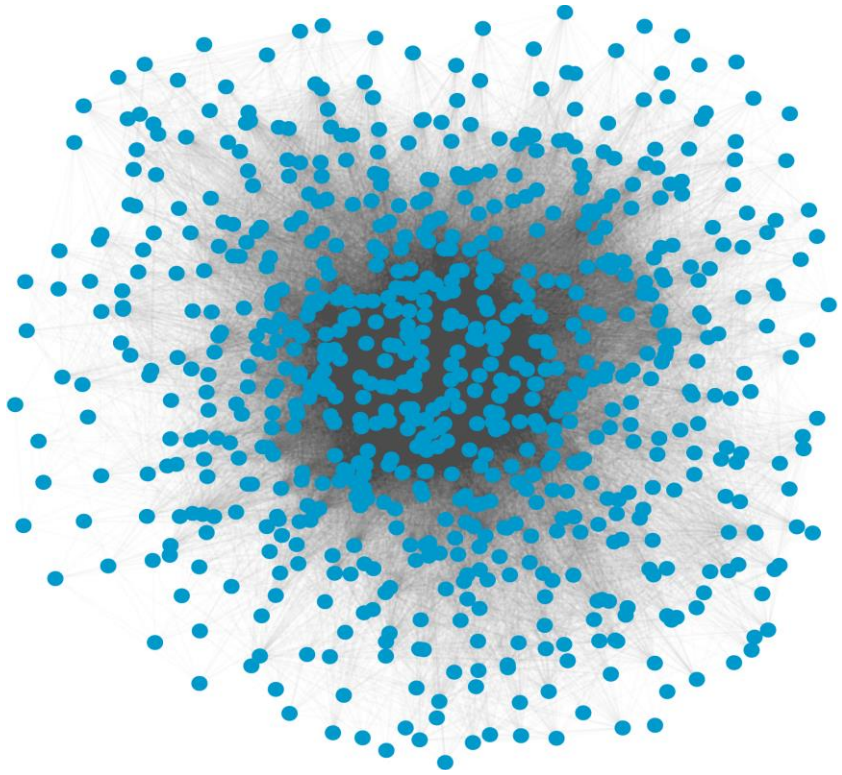

Supplementary Figure 1. CircRNA crosstalk (CC) network, the blue nodes represent circRNAs.

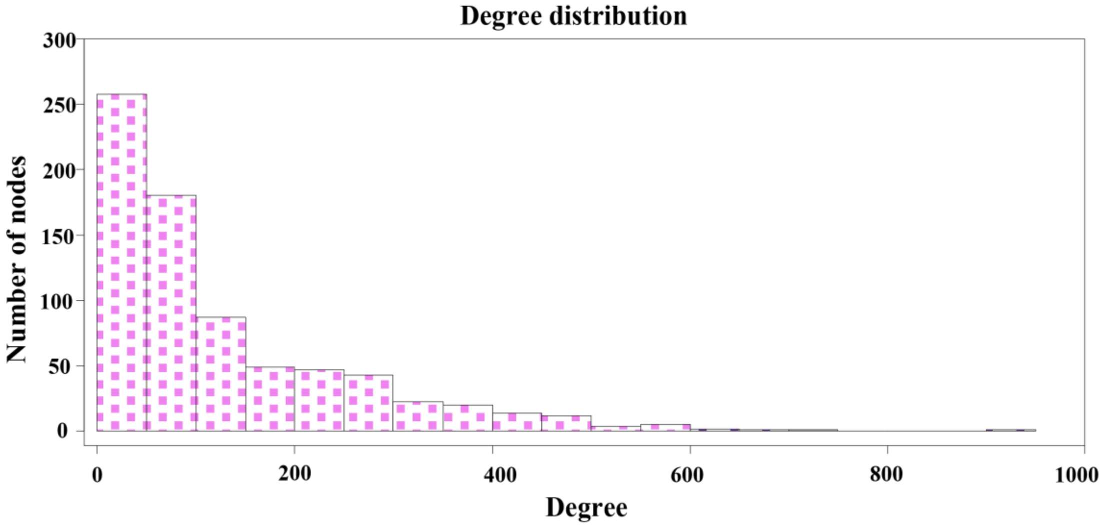

Supplementary Figure 2. Degree distribution of circRNA crosstalk (CC) network. The x-axis represents the degree of the network nodes, and y-axis represents the number of nodes.

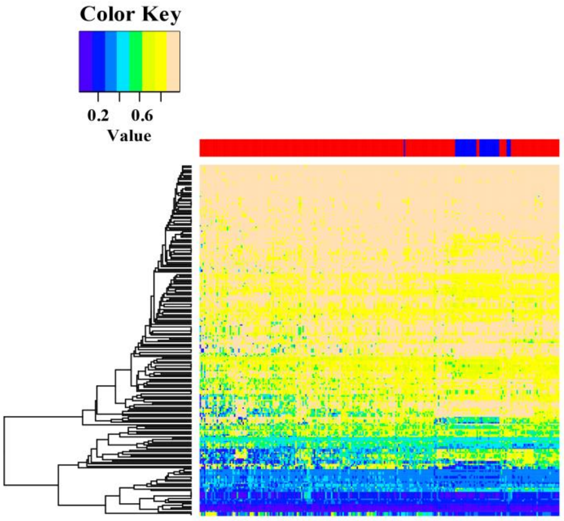

Supplementary Figure 3. The DNA methylation cluster analysis of differential methylation circRNA host genes between breast cancer samples and normal samples (red represents breast cancer samples, blue represents normal samples).

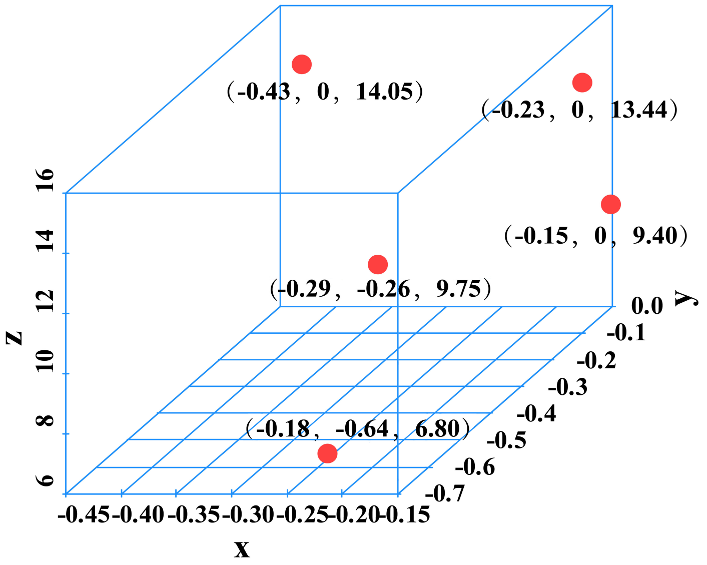

Supplementary Figure 4. A three-dimensional scatter plot represents that the co-regulation effect of DNA methylation of circRNA host genes and DNA methylation of driver genes on driver gene expression. The x-axis represents the Pearson correlation of DNA methylation of the driver gene and drive gene expression, the y-axis represents the Pearson correlation of the DNA methylation of the circRNA host gene and drive gene expression, and the z-axis represents the expression of the driver gene (Gene expresssion values were log2 transformed after adding a pseudo-value of 1 to avoid infinite values).

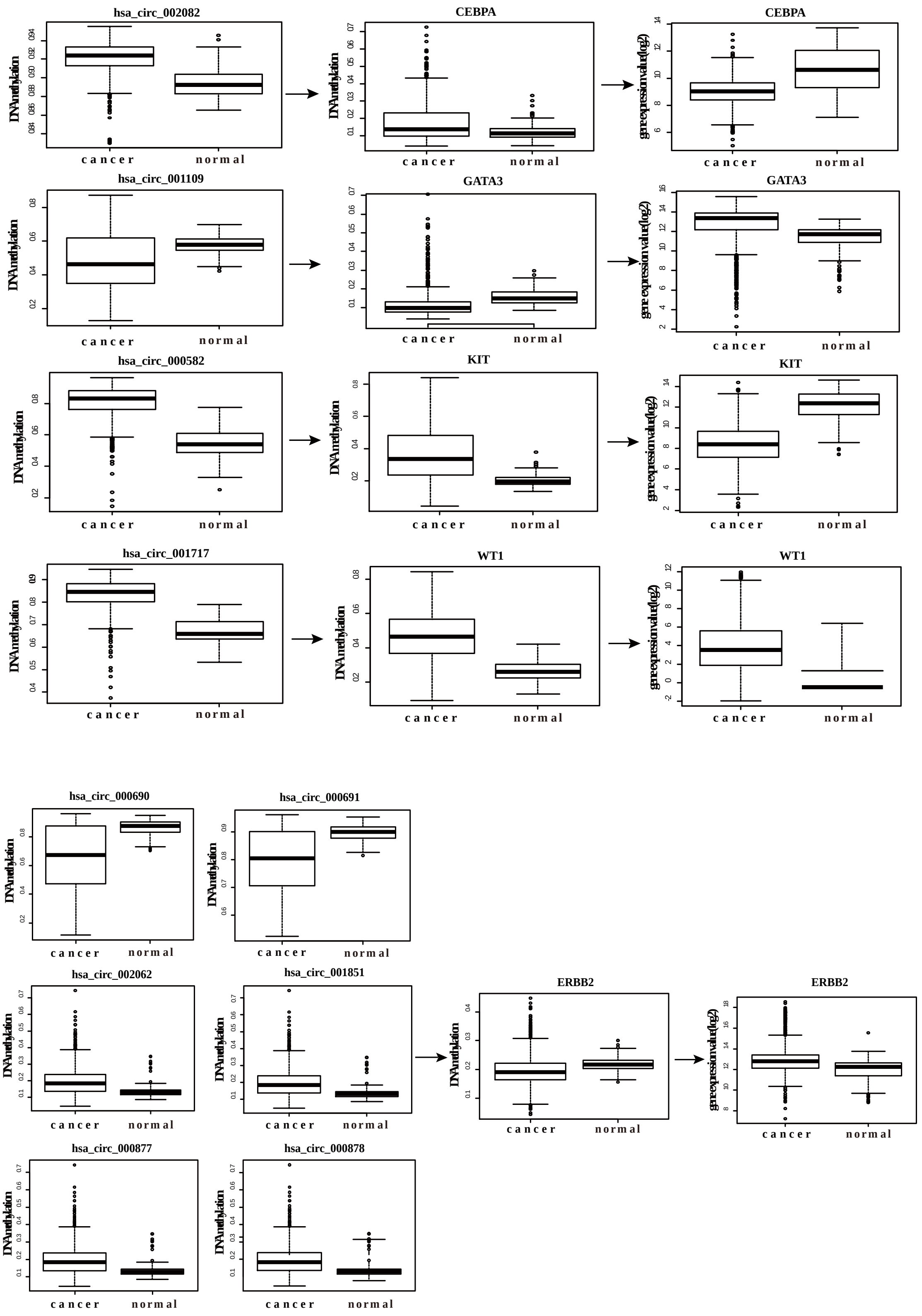

Supplementary Figure 5. The difference of DNA methylation of sponge circRNA host gene, DNA methylation of sponge driver gene and sponge driver gene expression between breast cancer samples and normal samples.

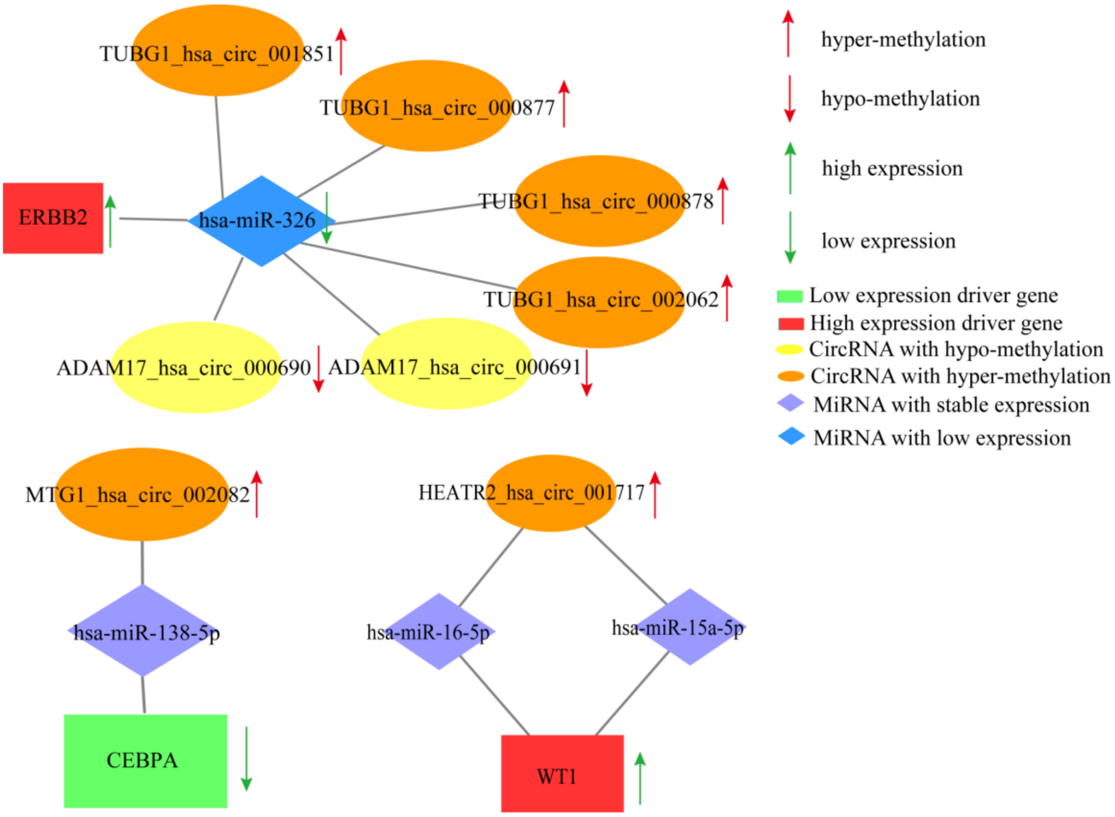

Supplementary Figure 6. Competition interaction diagram of ERBB2, CEBPA, WT1 and their sp_circRNAs.

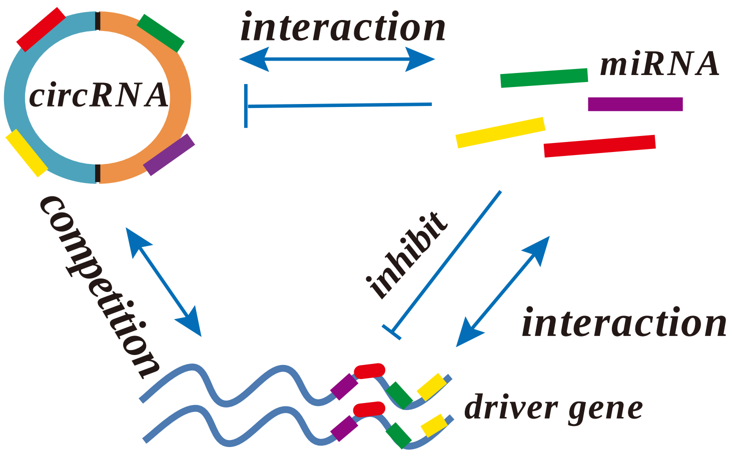

Supplementary Figure 7. The competition mechanism pattern diagram of sp_circRNA, miRNA and driver gene.
